# Tailoring Antithrombotic Regimens for Percutaneous Coronary Intervention Patients with High Bleeding and Ischemic Risk (*TAILOR-BIRISK*): Individualized Management and Genotype-Guided De-escalation

**DOI:** 10.31083/j.rcm2412348

**Published:** 2023-12-12

**Authors:** Junyan Zhang, Zhongxiu Chen, Yong He

**Affiliations:** ^1^Department of Cardiology, West China Hospital of Sichuan University, 610041 Chengdu, Sichuan, China

**Keywords:** percutaneous coronary intervention, high bleeding risk, high ischemic risk, genotype-guided, *CYP2C19*

## Abstract

Percutaneous coronary intervention (PCI) is a widely used reperfusion strategy for coronary artery disease, with millions of procedures performed annually. Attention has recently been drawn to a unique population, known as “*bi-risk*” patients, who have high ischemic and high bleeding risks and undergo PCI. However, there is currently no established definition or optimal antithrombotic therapy for this group. Genotype-guided antithrombotic therapy, which uses cytochrome *P450 (CYP) 2C19* gene testing, may offer a more personalized and precise approach. Nevertheless, recent research has shown that routine genetic testing to guide treatment in the PCI population does not improve patient outcomes, preventing it from being routinely recommended in guidelines. This review proposes, for the first time, the definition of the *bi-risk* population and the concept of *TAILOR-BIRISK* for their treatment strategies. *TAILOR-BIRISK* emphasizes de-escalating antithrombotic treatment and suggests that a short course of dual antiplatelet therapy (DAPT) followed by monotherapy by either clopidogrel or ticagrelor 60 mg BID (BID, twice daily) could be a reasonable option for this population. Additionally, the use of *CYP2C19* gene testing to guide ${\mathrm{P2Y}}_{\text{12}}$ inhibitor selection can help better individualize and customize the antithrombotic regimen. However, more large-sample randomized control studies should be conducted to further explore the optimal antithrombotic strategy for the *bi-risk* population.

## 1. Introduction

As the most widely used reperfusion therapy, percutaneous coronary intervention (PCI) is performed millions of times each year worldwide. Among them, the population with high bleeding or ischemic risk who also undergo PCI has received particular attention, as this population requires individual antithrombotic intensity and duration. A more unique population, namely the group with both high ischemic and high bleeding risk (*bi-risk*), has gradually begun to draw attention [1]. Currently, there is a lack of unified definition for this population, and the optimal antithrombotic strategy for this unique group has not previously been suggested. Therefore, we have conducted a comprehensive review of the current research on the *bi-risk* population and here propose our definition of *bi-risk* to increase awareness of this population.

Meanwhile, with the improvement in stent design quality and the control of high ischemic metabolic factors such as diabetes and hyperlipidemia, the overall ischemic event rate in patients undergoing PCI has significantly decreased, and the concept of de-escalation antithrombotic treatment has become prevalent. Current guidelines recommend that for patients with both high bleeding risk (HBR) and stent implantation, dual antiplatelet therapy (DAPT) is recommended for at least 6 months for acute coronary syndrome (ACS), while for chronic coronary syndrome (CCS) DAPT can be shortened to 1–3 months [2]. Several studies, such as prospective randomized comparison of the biofreedom biolimus A9 drug-coated stent versus the gazelle BMS (bare metal stent) in patients at high bleeding risk (LEADERS-FREE) and a randomized controlled trial with resolute onyx in one month DAPT for high-bleeding risk patients (ONYX ONE), have revealed that when using a new generation of drug-eluting stents in the HBR population, compared with bare-metal stents, it is safe and feasible to shorten the duration of DAPT to 1 month [3, 4]. In addition, scholars have also explored the feasibility of de-escalation antiplatelet therapy in patients with high ischemic risk. The short and optimal duration of dual antiplatelet therapy after everolimus-eluting cobalt-chromium stent (STOPDAPT-2) and STOPDAPT-2 ACS studies have gradually revealed the feasibility of maintenance treatment with clopidogrel after short-term DAPT [5, 6]. Therefore, we might consider a short duration of DAPT followed by clopidogrel monotherapy a suitable antithrombotic strategy for *bi-risk* patients. Additionally, the ticagrelor with aspirin or alone in high-risk patients after coronary intervention unfractionated heparin (TWILIGHT) series of studies have revealed that the use of ticagrelor in combination with aspirin for 3 months followed by monotherapy with ticagrelor in PCI patients is not inferior to 12 months of combination therapy with aspirin and ticagrelor [7]. Combined with the conclusion of the previous prevention of cardiovascular events in patients with prior heart attack using ticagrelor compared to placebo on a background of aspirin-thrombolysis in myocardial infarction 54 (PEGASUS-TIMI 54) study and the landmark analysis of the a clinical study comparing two forms of anti-platelet therapy after stent implantation (GLOBAL LEADERS) trial, we presume that the use of ticagrelor 60 mg BID (BID, twice daily) in the chronic maintenance period is also a feasible option for *bi-risk* populations [8, 9]. In addition, we reviewed studies on *CYP2C19* gene testing and speculated that testing drug metabolism genes could better help us develop the optimal antithrombotic regimen for the *bi-risk* population. We summarize our hypothesis above as “*TAILOR-BIRISK*”, an individualized, tailored de-escalation antithrombotic regimen for *bi-risk* populations.

This review proposes a definition of the *bi-risk* population and the concept of *TAILOR-BIRISK*, and we suggest that more large-sample randomized control studies should be conducted to further explore the optimal antithrombotic strategy for the *bi-risk* population.

## 2. Clinical Characteristics of *bi-risk* PCI Patients and Antithrombotic Treatment Strategy

### 2.1 Definition and Development of a bi-risk Group

#### 2.1.1 High Ischemic Risk Criteria and Development

In the early 1990s, studies demonstrated that both longer lesion lengths and longer stent lengths increased the incidence of ischemic events in patients after PCI. In the study by Kobayashi *et al*. [10], occurrence versus non-occurrence of in-stent restenosis in patient stent lengths were (32.8 ± 19.9) mm and (25.1 ± 14.8) mm (*p* < 0.001), respectively. Subsequently, data from the Dutch Stent Thrombosis Registry showed that in addition to longer stent lengths, factors such as multiple branch lesions and bifurcation lesions were also independent risk factors for in-stent thrombosis [11].

Coronary artery chronic total occlusion (CTO) lesions are generally defined as obstructive coronary artery lesions with positive thrombolysis in myocardial infarction (TIMI) flow grade 0 with occlusion time ≥3 months [12]. The process of CTO lesion management is complicated and often leads to pathophysiological mechanisms such as delayed healing of vascular injury, impaired coronary intima-media structure, and long-term endothelial dysfunction, which in turn causes platelet activation, aggregation, and thrombosis [13]. Therefore, CTO lesions are considered to be high risk ischemic lesions.

Cardiovascular interventionalists believe that the complexity of the PCI procedure is directly related to the patient’s subsequent adverse ischemic outcome [14]. Giustino *et al*. [15] pooled patient-level data from 6 randomized controlled trials and found that at a median follow-up time of 392 days, patients undergoing complex PCI had a higher risk of major adverse cardiovascular events (MACE) compared with non-complex PCI (hazard ratio [HR] 1.98, 95% confidence interval [CI] 1.50–2.60, *p* < 0.0001); and a significantly higher incidence of coronary thrombosis (HR 2.36, 95% CI 1.70–3.22, *p* < 0.0001). Complex PCI in this study was defined as PCI with one of the following characteristics: ≥3 stents placed, total stent length >60 mm, 2 stents placed in bifurcation lesions or PCI for CTO lesions.

Researchers also discovered that extracardiac diseases, including diabetes and chronic kidney disease, were significant risk factors for ischemia in patients after PCI. Diabetic patients are especially vulnerable to severe vasculopathy, as well as metabolic abnormalities like hyperglycemia and hyperinsulinemia, which can enhance platelet adhesion, activation and aggregation, ultimately leading to platelet hyperreactivity [16]. Likewise, chronic kidney disease (CKD) can contribute to an increased incidence of thrombotic events in post-PCI patients through a variety of mechanisms, such as intimal damage, accelerated vascular sclerosis, increased platelet aggregation and dyslipidemia. Subsequently, the 2018 European Society of Cardiology (ESC)/European Association for Cardio-Thoracic Surgery (EACTS) guidelines on myocardial revascularization proposed criteria for people at high ischemic risk (HIR), which included complex lesions such as diffuse lesions, bifurcation lesions, long lesions, CTO lesions, and other diseases such as diabetes and CKD [17]. The development of this standard will help clinicians manage this group of patients more accurately.

#### 2.1.2 High Bleeding Risk Criteria and Development

Similar to HIR, high bleeding risk (HBR) was also significantly associated with adverse events in patients undergoing PCI. Several bleeding risk stratification models have been developed to predict bleeding outcomes over different timeframes, from in-hospital to long-term [18, 19, 20, 21, 22, 23, 24, 25]. In the past, PCI was performed with femoral access and heparin plus glycoprotein inhibitors (GPIs) were administered during the procedure without considering stent types and activated clotting time [26, 27, 28]. As a result, bleeding incidents during PCI became a recurrent issue. Clinical trials primarily concentrated on minimizing the occurrence of major bleeding while patients were hospitalized. The initial bleeding risk stratification model, known as randomized evaluation in PCI linking angiomax to reduced clinical events (REPLACE-2), was documented in 2007 [29]. It was developed from two large, systematically collected datasets and served as a clinically useful risk model for in-hospital major bleeding. The modified model, which used only five preprocedural clinical variables, was capable of differentiating patients with low, moderate, and high risk of major bleeding. In 2009, Subherwal *et al*. [21] utilized data from the Can Rapid Risk Stratification of Unstable Angina Patients Suppress ADverse Outcomes with Early Implementation of the ACC/AHA Guidelines (CRUSADE) Quality Improvement Initiative to create and validate a scoring model for assessing the risk of major bleeding during hospitalization in patients with non-ST-segment elevation myocardial infarction. They consolidated eight baseline factors into a straightforward and validated tool known as the CRUSADE score [21].

Improvements in bleeding prevention techniques have led to a decrease in the occurrence of in-hospital bleeding incidents over time. Contemporary predictive models now anticipate the likelihood of out-of-hospital bleeding [30, 31]. The predicting bleeding complications in patients undergoing stent implantation and subsequent dual antiplatelet therapy (PRECISE-DAPT) score incorporates five factors to forecast the probability of out-of-hospital bleeding within one year. According to Costa *et al*. [24], patients with a PRECISE-DAPT score of 25 or higher, indicating a high risk of bleeding, are advised to follow a shortened DAPT regimen of less than 12 months. This recommendation is further supported by the post hoc analysis of 6-month versus 12-month or longer dual antiplatelet therapy after percutaneous coronary intervention in patients with acute coronary syndrome (SMART-DATE), which highlights the benefits of a shortened 6-month DAPT regimen for patients with a PRECISE-DAPT score of 25 or above [32]. In 2019, the Academic Research Consortium for High Bleeding Risk (ARC-HBR) established a consensus definition of high bleeding risk based on the available evidence [33]. The 2020 ESC Guidelines for the management of acute coronary syndromes in patients without persistent ST-segment elevation have endorsed the utilization of two bleeding risk stratification tools, namely PRECISE-DAPT and ARC-HBR, to determine the optimal duration of dual antiplatelet therapy [34].

### 2.2 Characteristics and Definitions of bi-risk Groups

The preceding exposition has delineated the definition and development of both HIR and HBR as well as their respective characteristics. However, for patients who exhibit both proclivities for bleeding and ischemia, they are designated as the *bi-risk* population. This group can be bifurcated into two subcategories based on the different risk factors they possess: (1) those individuals who inherently exhibit factors that elevate both bleeding and thrombotic risks, such as chronic kidney disease, advanced age, ischemic stroke, and so forth; and (2) those who have both HIR and HBR risk factors. To date, there is no standardized definition that clearly delimits this population, but one study’s protocol has proposed their definition of *bi-risk* patients [1]. We have combined their definition with our collection and integration of existing evidence to present our own definition of *bi-risk* patients, as shown in Table 1 (Ref. [1]). This population is not uncommon and necessitates judicious selection and management to balance their competing risks of bleeding and thrombosis.

**Table 1.** **Definition of *bi-risk* population [1]**.

Definition of *bi-risk* patients			
The *bi-risk* population is a group of patients who are evaluated for both high bleeding and high ischemia at the time of PCI. Under these conditions any one of the two following criteria meets the diagnosis for *bi-risk*:			
Patient meets at least one of the following criteria for both high bleeding and high ischemia:			
	Chronic kidney disease (defined as an estimated glomerular filtration rate <60 mL/min/1.73 $m^{\text{2}}$)		
	Elderly patients (defined as age >75 years)		
	Moderate or severe ischemic stroke within the past 6 months		
Patient meets at least one high ischemic risk factor and at least one major high bleeding risk factor or two minor high bleeding risk factors at the same time:			
	High bleeding risk criteria		
		Main criteria	
			Anticipated use of long-term oral anticoagulation
			Hemoglobin <11 g/dL
			Spontaneous bleeding requiring hospitalization or transfusion in the past 6 months or at any time, if recurrent
			Moderate or severe baseline thrombocytopenia (platelet count <100 × ${\mathrm{10}}^{\text{9}}$/L)
			Chronic bleeding diathesis
			Liver cirrhosis with portal hypertension
			Active malignancy (excluding nonmelanoma skin cancer) within the past 12 months
			Previous spontaneous intracranial hemorrhage (at any time)
			Nondeferrable major surgery on dual antiplatelet therapy
			Recent major surgery or major trauma within 30 days before PCI
		Secondary criteria	
			Hemoglobin 11–12.9 g/dL for men and 11–11.9 g/dL for women
			Spontaneous bleeding requiring hospitalization or transfusion within the past 12 months not meeting the major criterion
			Long-term use of oral NSAIDs or steroids
	High ischemic risk criteria		
			Acute coronary syndrome
			Multiple coronary lesions
			Target lesions with a total stent length greater than 30 mm
			Thrombotic target lesions
			Left main (≥50%) or proximal anterior descending (≥70%) lesion
			Bifurcation lesion (Medina staging 0, 1, 1, or 1, 1, 1) requiring at least 2 stents
			Calcified target lesion requiring rotational atherectomy
			Concomitant with defined vascular disease
			Chronic total occlusion
			Recurrent myocardial infarction, coronary revascularization, in-stent thrombosis, stroke within 9 months prior to PCI
			Concomitant with diabetes requiring medication

PCI, percutaneous coronary intervention; NSAIDs, nonsteroidal anti-inflammatory drugs.

### 2.3 Antithrombotic Therapy in Patients with bi-risk

DAPT consisting of aspirin and a ${\mathrm{P2Y}}_{\text{12}}$ receptor inhibitor serves as the foundation of pharmacological treatment for patients post-PCI. It aims to prevent post-procedure thromboembolic events, such as stent thrombosis and re-infarction, by suppressing platelet aggregation.

Since the definition of ARC-HBR proposed in 2019, studies have focused extensively on PCI-HBR population. Subsequent research such as management of high bleeding risk patients post bioresorbable polymer coated stent implantation with an abbreviated versus prolonged DAPT regimen (MASTER-DAPT) [35], TWILIGHT-HBR [7], evaluate safety and effectiveness of the tivoli drug-eluting stents (DES) and the firebird DES for treatment of coronary revascularization (I LOVE-IT-2) [36] and STOPDAPT-2 [37], all suggest that PCI-HBR patients have a higher risk of bleeding and a worse prognosis, and attenuating antithrombotic regimens in this group of patients can improve the prognosis of HBR patients, by greatly reducing the risk of bleeding, without increasing the risk of ischemia. The MASTER-DAPT study involved 4579 patients with HBR who underwent PCI using biodegradable polymeric sirolimus-eluting stents. The study concluded that a one-month DAPT regimen was equally effective as three months or more of DAPT, and even showed a reduced occurrence of major or clinically relevant non-major bleeding [35]. Similarly, the STOPDAPT-2 study demonstrated that shortening DAPT to one month, followed by clopidogrel monotherapy, did not lead to an increase in ischemic events. On the contrary, it resulted in a decrease in major bleeding events, particularly benefiting the HBR subgroup [37].

**Fig. 1.**
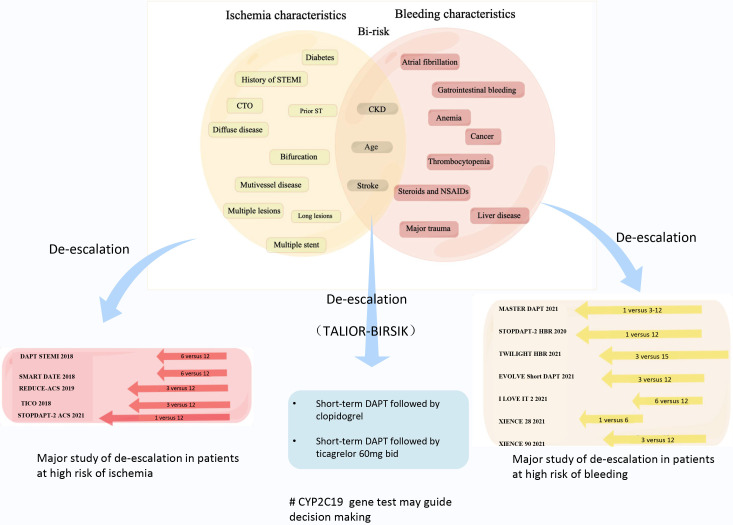
**Characteristics of patients undergoing PCI with high bleeding and ischemia risk and the concept of *TAILOR-BIRISK***. The concept indicates de-escalating antithrombotic treatment and suggests that short-term dual antiplatelet therapy combined with clopidogrel monotherapy or ticagrelor 60 mg BID monotherapy in maintenance is feasible for this *bi-risk* population. The use of *CYP2C19 *gene testing to guide ${\mathrm{P2Y}}_{\text{12}}$ inhibitor selection can help better individualize and customize the antithrombotic regimen. STEMI, ST segment elevation myocardial infarction; CTO, chronic total occlusion; CKD, chronic kidney disease; NSAIDs, nonsteroidal anti-inflammatory drugs; *CYP2C19*, cytochrome P*450 2C19*; ST, stent thrombosis; DAPT, dual antiplatelet therapy; SMART-DATE, smart angioplasty research team-safety of 6-month duration of dual antiplatelet therapy after percutaneous coronary intervention in patients with acute coronary syndrome; REDUCE, short-term dual anti platelet therapy in patients with ACS treated with the COMBO dual-therapy stent; TICO, ticagrelor monotherapy after 3 months in the patients treated with new generation sirolimus stent for acute coronary syndrome; STOPDAPT-2, the short and optimal duration of dual antiplatelet therapy after everolimus-eluting cobalt-chromium stent; ACS, acute coronary syndrome; MASTER DAPT, management of high bleeding risk patients post bioresorbable polymer coated stent implantation with an abbreviated versus prolonged DAPT regimen; HBR, high bleeding risk; EVOLVE, a prospective, multicenter, single-arm study designed to assess the safety of 3-month DAPT in subjects at high risk for bleeding undergoing PCI with the SYNERGY everolimus-eluting platinum chromium coronary stent system; TWILIGHT, ticagrelor with aspirin or alone in high-risk patients after coronary intervention unfractionated heparin; I LOVE IT 2, evaluate safety and effectiveness of the tivoli DES and the firebird DES for treatment of coronary revascularization; PCI, percutaneous coronary intervention; BID, twice daily; DES, drug-eluting stents.

Research has unveiled that individuals belonging to the PCI-HBR patient cohort often face a heightened likelihood of encountering ischemic events [38, 39, 40]. Given the aging of the global population, patients with coronary artery disease (CAD) are increasingly presenting with comorbidities, more complex coronary lesions, and more severe disease. Features associated with a high risk of ischemia, such as multi-vessel disease, diffuse disease, bifurcation lesions, calcified lesions, long lesions, recurrent myocardial infarctions, as well as comorbidities such as diabetes and renal dysfunction, are becoming more prevalent in patients. These high ischemic factors, in turn, make this group of patients require more potent antithrombotic regimens to control the significantly elevated ischemic events [41]. For CAD patients with both a high risk of bleeding and a high risk of ischemia, it is crucial to exercise caution in selecting their antiplatelet therapy. An overly aggressive approach may increase the risk of bleeding, while an approach that is too weak may lead to ischemic events such as myocardial infarction and in-stent thrombosis. Therefore, the *bi-risk* population has been defined to raise awareness and assist cardiologists in better managing these special individuals [1]. Thus, given the evidence regarding antiplatelet strategy above, we propose for the first time the concept of *TAILOR-BIRISK* for the treatment of the *bi-risk* population undergoing PCI (Fig. 1). We emphasize that a short course of DAPT in combination with clopidogrel monotherapy is feasible for this population, and that short-term DAPT combined with maintenance treatment with ticagrelor 60 mg BID is also feasible. *TAILOR-BIRISK* also impliesthat the use of the *CYP2C19* gene test to guide de-escalating strategies can help to better individualize and customize the anti-thrombotic regimen.

However, no study so far has directly investigated the possibility of reducing ticagrelor to 60 mg BID as monotherapy during the first 12 months after PCI, and there is a lack of large randomized controlled trials that compare various antithrombotic regimens in *bi-risk *patients. The ongoing optimal antiplatelet therapy for high bleeding and ischemic risk patients (OPT-BIRISK) Trial (NCT 03431142) compares the use of extended DAPT for maintenance treatment versus clopidogrel monotherapy after 9–12 months of DAPT; and is anticipated to provide new evidence for antithrombotic therapy during the chronic maintenance period for *bi-risk* patients [1]. However, given that the peri-PCI and early post-PCI periods are high-risk times for in-stent thrombosis and reinfarction, it remains essential to study the best antithrombotic strategy during this critical period. Therefore, more research should be focused on the peri-PCI stage and the early stages following PCI in *bi-risk* patients.

### 2.4 The Value of Genotype-Guided Treatment in Antithrombotic Therapy for PCI

**Fig. 2.**
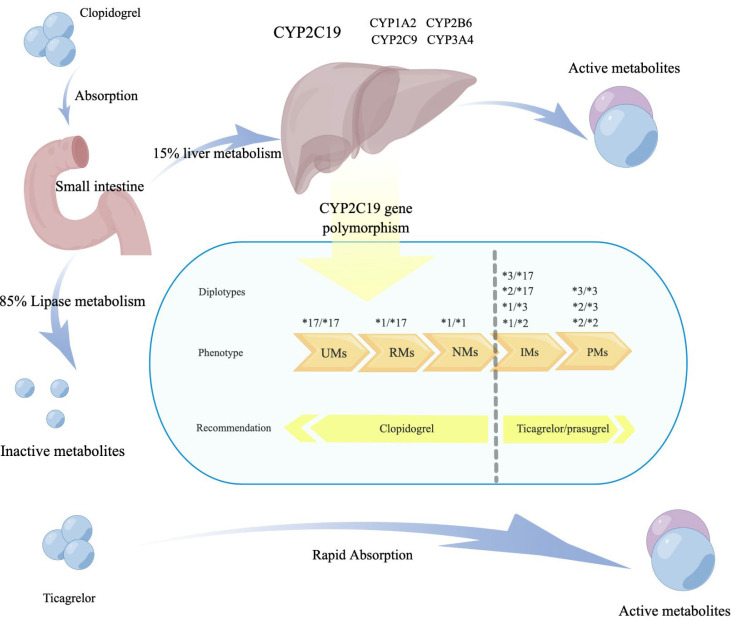
**Metabolism of clopidogrel and the impact of different genotypes on drug recommendations**. CYP, cytochrome P450; UMs, ultrarapid metabolizers; RMs, rapid metabolizers; NMs, normal metabolizers; IMs, intermediate metabolizers; PMs, poor metabolizers.

Research has confirmed that the risk of cardiovascular adverse events in individuals taking clopidogrel is directly related to their *CYP2C19 *gene polymorphism, as the *CYP2C19* enzyme encoded by the gene is involved in the metabolism of clopidogrel [42]. Clopidogrel itself is an inactive prodrug, and about 85% of it is hydrolyzed into inactive metabolites by esterases in the small intestine after absorption, while only about 15% of clopidogrel is metabolized in the liver through the *CYP2C19*-mediated process to form active metabolites that irreversibly bind to the platelet membrane surface adenosine diphosphate (ADP) receptor ${\mathrm{P2Y}}_{\text{12}}$, inhibiting ADP-mediated platelet activation and aggregation [43] (Fig. 2). Therefore, *CYP2C19* enzyme activity determines the metabolic efficiency of clopidogrel in patients.

The *CYP2C19 *gene has polymorphisms, and the polymorphisms at some loci can affect enzyme activity, ultimately leading to differences in the metabolic efficiency of drugs in different individuals. In large-scale meta-analyses, it has been demonstrated that patients receiving clopidogrel treatment during PCI who possess the *CYP2C19* intermediate metabolizer (IM) genotype, such as *1/*2 and *1/*3, or the poor metabolizer (PM) genotype, such as *2/*2 and *2/*3, exhibit an elevated risk of experiencing MACE and stent thrombosis compared to those with the normal metabolizer (NM) genotype, i.e, *1/*1 [44, 45, 46].

A multicenter randomized controlled trial by Cavallari *et al*. [47] showed a higher incidence of MACE after PCI in patients with *LoF* gene variants treated with clopidogrel. After 1 year of follow-up, MACE was more likely to occur in the clopidogrel-treated group compared with other treatment groups amongst *CYP2C19 LoF* allele carriers (adjusted hazard ratio (HR) 2.21, *p* = 0.021). For patients receiving prasugrel or ticagrelor, the risk of MACE was similar in the *CYP2C19 LoF* allele group compared with the no-*LoF* allele group (adjusted HR 0.81, 95% CI 0.48–1.35; *p* = 0.41) [47]. Similarly, a meta-analysis of randomized control trials suggested a higher risk of MACE in patients with *CYP219 LoF *gene variants when treated with clopidogrel compared with other ${\mathrm{P2Y}}_{12}$ inhibitors (risk ration (RR) 1.42, 95% CI 1.2–1.7). For patients without *LoF gene *variants, the risk of cardiovascular events was similar for clopidogrel versus other ${\mathrm{P2Y}}_{12}$ inhibitor therapy (RR 1.0, 95% CI 0.8–1.25) [48]. Therefore, the theories above provide the basis for genotype-guided treatment regimens when considering patients treated with clopidogrel.

However, multiple studies aiming to demonstrate the superiority of genotype-guided antithrombotic strategies have not achieved the expected results. A meta-analysis by Bauer *et al*. [49] including 15 randomized controlled trials (RCTs) did not support routine *CYP2C19* gene testing of patients to guide platelet therapy. Published in 2020, the Tailored Antiplatelet Initiation to Lesson Outcomes Due to Decreased Clopidogrel Response after Percutaneous Coronary Intervention study (TAILOR-PCI) is the largest study using *CYP2C19* genetic testing to guide antiplatelet treatment strategies after PCI, with 5302 PCI patients randomized to a genotype-guided strategy group and a conventional treatment strategy group and followed for 12 months. In the genotype-guided treatment group, ticagrelor was given to *CYP2C19 LoF* allele carriers and clopidogrel to wild-type allele carriers; all patients in the standard treatment group received clopidogrel. However, the results of the study showed that a genotype-directed post-PCI DAPT regimen did not significantly reduce adverse cardiovascular events. The difference in the composite endpoint of cardiovascular death, myocardial infarction, stroke, in-stent thrombosis, and serious recurrent ischemic events at 12 months was not statistically significant for the primary endpoint of genotype-guided versus conventional treatment strategy (4.03% vs. 5.85%, OR = 0.66, *p* = 0.056) [50]. Similarly, the genotype-guided treatment group in the Patient Outcome After Primary PCI (POPular) Genetics study did not significantly reduce the net clinical endpoint events compared with the conventional treatment group (5.1% vs. 5.9% in the standard treatment group; absolute rate difference 0.7%; 95% CI 2.0–0.7) [51]. All patients in the standard treatment arm of the study received prasugrel or ticagrelor. This trial also suggests that in high ischemic risk patients, genotype-guided ${\mathrm{P2Y}}_{\text{12}}$ inhibitor selection is not inferior to the routine use of potent ${\mathrm{P2Y}}_{\text{12}}$ inhibitors.

However, multiple guidelines and expert consensus currently do not routinely endorse genetic testing to inform antiplatelet therapy after PCI due to several reasons [34, 52, 53]. Firstly, there is inadequate evidence demonstrating that genetic testing can substantially enhance the efficacy and safety of antiplatelet therapy after PCI. Secondly, genetic testing may escalate treatment expenses and necessitate additional time and resources, which may not prove to be cost-effective. Thirdly, the therapeutic efficacy of clopidogrel is predominantly governed by the *CYP2C19* gene, although other genetic polymorphisms such as *ABCB1* and *PON1*, as well as the overall physiological function of the body, also play a role [54, 55]. This elucidates why gene-guided antiplatelet therapy may confer greater benefit to patients in the early post-PCI period (post-procedure to 3–6 months), whereas this benefit tends to diminish during the chronic maintenance phase. Moreover, the *CYP2C19* test can only indicate possible metabolic types of clopidogrel instead of actual efficacy, and combined platelet function tests may be possible to better assess the actual effects of clopidogrel. However, platelet function tests require continuous dynamic monitoring, unlike genetic testing, which is a one-time test. Clinically, for high-risk ischemic populations, direct use of ticagrelor is often preferred rather than testing clopidogrel metabolism to guide drug use.

Although most guidelines do not routinely recommend genetic testing for patients requiring clopidogrel after PCI, some make recommendations for specific populations. The 2017 ESC guideline recommended that patients with recurrent adverse events may undergo genetic testing to guide patients on the need to change antiplatelet agents [2].

The 2022 position statement from the European Society of Cardiology Working Group on Cardiovascular Drug Therapy recommended that consideration should be given to genotyping high-risk cardiovascular patients, especially those at high risk of thrombosis or bleeding, before prescribing clopidogrel [56].

Currently, the majority of evidence suggests that routine *CYP2C19* genetic testing in the PCI population is not feasible. However, the value of such testing may still be untapped in certain special populations, such as patients with high ischemic or bleeding risk, and further research in this area is needed.

### 2.5 The Promising Potential of Genotype-Guidance in a bi-risk Patient Population

As the second generation of drug-eluting stents feature unique technologies such as thin strut design and ultra-thin biodegradable polymer coating that aids rapid endothelialization and reduces the sustained risk of stent thrombosis; coupled with the recent emphasis on intensive blood pressure and lipid-lowering interventions targeting coronary risk factors, the long-term thrombotic risk has decreased compared to the past and de-escalation has therefore become a research hotspot in the field of antithrombotic therapy for PCI in recent years [57, 58]. Currently, the optimal thrombotic method for PCI in *bi-risk* patients remains uncertain. The trend of de-escalating antithrombotic therapy in patients with high risk of ischemia or bleeding indicates that it may be equally feasible to shorten the duration of DAPT in patients with *bi- risk*. From existing studies targeting patients separately with HBR or HIR, it appears that a de-escalation approach using short-term DAPT followed by a ${\mathrm{P2Y}}_{\text{12}}$ inhibitor alone is a research trend for future antithrombotic strategies in *bi-risk* patients undergoing PCI [6, 59, 60, 61].

While aspirin has been the preferred antiplatelet drug for CAD based on its long history and extensive clinical research, recent studies such as aspirin versus clopidogrel for chronic maintenance monotherapy after percutaneous coronary intervention (HOST-EXAM) study [62] and HOST-EXAM extended study [63] suggest that ${\mathrm{P2Y}}_{\text{12}}$ inhibitors may have the potential to surpass aspirin in the chronic maintenance phase. For instance, the HOST-EXAM study demonstrated that compared to aspirin, clopidogrel not only reduces the risk of ischemic events but also decreases the risk of bleeding in the chronic maintenance phase. This combination of efficacy and safety benefits aligns with the needs of *bi-risk* patients. Therefore, we propose that ${\mathrm{P2Y}}_{\text{12}}$ receptor inhibitors may be a more favorable choice for maintenance therapy in *bi-risk* patients.

Roule *et al*. [64]’s meta-analysis provides evidence for this hypothesis. They included a specific *bi-risk* population, elderly patients, and demonstrated that the use of ${\mathrm{P2Y}}_{\text{12}}$ inhibitors after short DAPT is a good option for these patients. Although elderly patients are not directly listed as a HIR criterion in the 2018 ESC guidelines, many studies have shown that elderly patients often have both high ischemic and high bleeding risks.

In the context of the increasing importance of ${\mathrm{P2Y}}_{\text{12}}$ receptor antagonists, personalized selection has become crucial. In *bi-risk* populations, routine use of clopidogrel without considering the presence of *CYP2C19 *genetic polymorphisms may increase the risk of ischemia due to clopidogrel resistance. Conversely, routine use of ticagrelor to address ischemic risk may increase the risk of major bleeding in these patients. Therefore, *CYP2C19* genetic testing to guide the selection of ${\mathrm{P2Y}}_{\text{12}}$ receptor antagonists in these *bi-risk* populations represents a promising approach.

For *bi-risk* patients, genetic testing can guide the selection of ${\mathrm{P2Y}}_{\text{12}}$ inhibitors during the peri-PCI period and early period after PCI; on the other hand, during the maintenance phase of antiplatelet therapy after short-term DAPT, there is lack of evidence to suggest whether *CYP2C19* genetic testing may guide the use of a reduced-dose ticagrelor strategy (i.e., 60 mg BID). Certain studies have suggested that chronic maintenance treatment with ticagrelor 60 mg BID is equally effective and safe compared with ticagrelor 90 mg BID for stable CAD patients with high ischemia risk, and there was a tendency towards lower rates of respiratory distress and major bleeding events [9]. Currently, there is a paucity of research investigating the comparative efficacy of reduced-dose ticagrelor versus clopidogrel in the chronic maintenance phase for *bi-risk patients* with the *CYP2C19 LoF* allele. Therefore, we believe that in the context of genetic guidance, ticagrelor 90 mg BID in short-term DAPT duration combined with maintenance therapy with reduced-dose ticagrelor (60 mg BID) may be a promising option for *bi-risk* patients carrying the clopidogrel *LOF* gene. However, there is a lack of direct research evidence to support this conclusion, and more studies will be needed to confirm it.

## 3. Conclusions and Clinical Perspectives

The optimal antithrombotic therapy for *bi-risk* patients undergoing PCI, who are at high risk for both ischemic and bleeding complications, remains unclear. This review proposes the definition of the *bi-risk* population and the concept of *TAILOR-BIRISK*, which implies that a short course of DAPT followed by monotherapy by either clopidogrel or ticagrelor 60 mg BID could be a reasonable option for this population. Nevertheless, genotype-guided antithrombotic therapy, which employs *CYP2C19 *gene testing, has shown potential in providing a more personalized and precise approach. Although the current limitations of genetic testing in terms of time and cost prevent it from being routinely recommended in guidelines, this review provides a summary of the latest evidence on *CYP2C19* gene testing and the characteristics of dual high-risk patients to fill the gap in evidence-based antithrombotic treatment. This approach has the potential to offer a more individualized approach to the *bi-risk* population, possibly assisting the choice between the two aforementioned proposed treatment strategies. More large-sample randomized control studies should be conducted to further explore the optimal antithrombotic strategy for the *bi-risk* population.
